# Desalination of Groundwater from a Well in Puglia Region (Italy) by Al_2_O_3_-Doped Silica and Polymeric Nanofiltration Membranes

**DOI:** 10.3390/nano10091738

**Published:** 2020-09-01

**Authors:** Xianzheng Ma, Cejna Anna Quist-Jensen, Aamer Ali, Vittorio Boffa

**Affiliations:** Center for Membrane Technology, Department of Chemistry and Bioscience, Aalborg University, Fredrik Bajers vej 7H, 9220 Aalborg, Denmark; xm@bio.aau.dk (X.M.); cejna@bio.aau.dk (C.A.Q.-J.); aa@bio.aau.dk (A.A.)

**Keywords:** membrane materials, surfactant templated-silica, drinking water, pesticides

## Abstract

Some of the groundwater aquifers in the Puglia Region, Italy, suffer from high salinity and potential micropollutant contamination due to seawater infiltration and chemical discharge. The objective of this study is twofold: to evaluate the performance of the recently reported alumina-doped silica nanofiltration membranes for water potabilization, and to provide a possible solution to improve the groundwater quality in the Puglia Region while maintaining a low energy-footprint. Two lab-made alumina-doped silica membranes with different pore structures, namely S/O = 0.5 and S/O = 2, were tested with real groundwater samples and their performances were compared with those of a commercial polymeric membrane (Dow NF90). Moreover, groundwater samples were sparked with acetamiprid, imidacloprid, and thiacloprid to test the membrane performance in the presence of potential contamination by pesticides. At a trans-membrane pressure of 5 bar, NF90 could reduce the groundwater conductivity from 4.6 to around 1.3 mS·cm^−1^ and reject 56–85% of the model pesticides, with a permeate flux of 14.2 L·m^−2^·h^−1^. The two inorganic membranes S/O = 2 and S/O = 0.5 reduced the permeate conductivity to 3.8 and 2.4 mS·cm^−1^, respectively. The specific energy consumption for all three membranes was below 0.2 kWh·m^−3^ which indicates that the potabilization of this groundwater by nanofiltration is commercially feasible.

## 1. Introduction

The shortage of clean water is one of the most pressing issues for humanity. Groundwater has always been an extremely valuable water resource, nowadays it contributes to around 30% of the total freshwater supply in the world [1]. However, minerals or organic pollutants might infiltrate aquifers, thus compromising the quality of groundwater. Indeed, a large amount of clean water supply in Europe comes from karst aquifers, which can be contaminated by natural or anthropogenic processes [2]. Over the years, there have been two emerging challenges for the groundwater in the Puglia Region, Italy. On the one hand, some of the aquifers have high salinity, due to the infiltration of seawater, which makes the groundwater unsuitable for human consumption [3]. On the other hand, the groundwater in the Puglia Region could be subjected to potential micropollutants contamination due to the excessive discharge of pesticides and pharmaceuticals [4]. Therefore, desalination and detoxification of the groundwater are vital for local water supplies.

Pressure-driven membrane processes such as reverse osmosis (RO) or nanofiltration (NF) can be applied to reduce the salinity. NF and RO membranes are commercially available and have proven to be effective for partial or full desalination treatments [3,4,5,6]. In RO membranes, the active layer consists of a dense polymer, through which water transport occurs via a solution-diffusion mechanism. In reason of their dense structure, these membranes can reach rejection for monovalent ions higher than 99% [7,8] but the RO membranes require high pressure for the operation (generally, about 15–30 bar for brackish water and 55–70 bar for seawater [9]), which implies high investment costs and high energy consumption during operation. On the contrary, the active layer of NF membranes is porous. In general, the pore size if NF membranes in the range 0.5–2 nm (around 0.5–2 kDa for the molecular weight cut-off) [10]. Therefore, NF membranes allow for higher flux compared to RO membranes. Rejection in NF membranes depends on three mechanisms: the steric exclusion of the nano-sized pores, the Donnan exclusion due to the ionization of the surface functional group, and the dielectric exclusion caused by the energy barrier when ions move from the bulk solution to the confined pores [11,12]. NF membranes can only partially reject monovalent ions, but they operate at a much lower pressure than RO systems and show high retention for the divalent ions, heavy metal ions, and organic pollutants. In reason of these features, NF membranes are more economically favorable than RO for those applications, which complete desalination is not required [13]. To date, most of the NF systems rely on polymeric membranes. However, the lifespan of the polymeric membranes might be compromised by mechanical damage and repetitive chemically cleaning [7,14]. In recent years, an increasing interest has emerged towards inorganic membranes since they are, in principle, more robust and durable than their polymeric counterpart [15,16,17]. Yet, the cost for the inorganic membranes could be much higher compare to the polymer membrane, but the high manufacturing cost could be compensated by its long lifespan [18]. Inorganic membranes such as silica membranes have shown great potential for filtration applications. Yet, pure silica membranes suffer from hydrothermal instability, meaning the membrane selectivity and permeability would deteriorating rapidly during filtration. Studies have shown the doping of the metal oxides, including alumina, could stabilize the silica membrane [19,20,21]. In a previous study, we reported new silica membranes doped with 5 mol% alumina, whose permeability and selectivity can be tuned by the concentration of a surfactant that acts as a pore-forming agent in the synthesis mixture [22]. Optimization of such membranes allowed to achieve water permeability higher than 2 L·(m^2^·h·bar)^−1^, rejection of around 95% for Mg^2+^ when tested with a model solution of MgSO_4_ (ionic strength = 0.01 M) and almost complete rejection for 10 ppm of caffeine [22]. Therefore, Al_2_O_3_-doped silica membranes have shown potential for water purification and detoxication, but their filtration performance in a real-life application has not been investigated yet.

In this context, the objective of this study is twofold. Firstly, the study aims to test the performances of the new Al_2_O_3_-doped silica membranes in comparison with a state-of-the-art commercial polymeric NF reference in a relevant case-study, namely the desalination and detoxification of the groundwater. Among the commercially available polymeric membranes, the NF series from Dow Filmtec has been widely studied [10,11,12]. NF90, in particular, shows a high rejection of salt ions, e.g., about 94–99% for Mg^2+^ [23,24,25]. Thus, here we selected this membrane as a commercial reference. Al_2_O_3_-doped silica membranes and NF90 were compared for their filtration performances and their specific energy consumption. Secondly, this study wishes to provide a possible desalination technology for a well owned by Acquedotto Pugliese S.P.A. (Puglia Region, Italy). The well is located near the river Galeso, at less than 3 km from the city of Taranto. This geographical area is characterized by water stress, due to a combination of moderate rainfall supply (<500 mm/year), high density of population (~200,000 inhabitants over an area of 250 km^2^), and an industrial district that includes the largest steel factory in Italy. The groundwater in this specific site has a pH of 7.5, and the concentration of pathogens or heavy metals are below the limits to be harmful for humans. However, the conductivity of the water is at 4.6 mS·cm^−1^, which needs to be reduced to 2.5 mS·cm^−1^ in order to be suitable for human consumption according to the Italian authorities [26]. The contamination of water resources by organic micropollutants has been reported in the region. Therefore, NF membranes performances were also tested with water samples spiked with model pollutants. 

## 2. Materials and Methods

### 2.1. Fabrication and Characterization of Al_2_O_3_-Doped Silica Membranes

**Figure 1:**
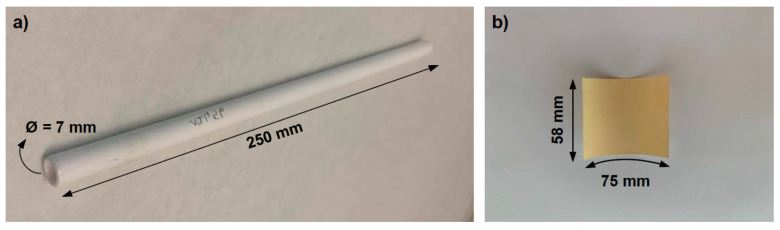
(**a**) Ceramic tube used as support for the Al_2_O_3_-doped silica nanofiltration (NF) membranes; (**b**) flat-sheet sample of the polymeric NF90 (FilmTec™) used in this study.

In this study, two 5 mol% Al_2_O_3_-doped silica membranes with different pore structure were fabricated via the sol-gel method, the detailed synthesized procedure was described in the previous study [22]. In brief, a two-steps sol-gel synthesis was applied. At first, tetraethyl orthosilicate (TEOS, 98%, Sigma Aldrich, St. Louis, MO, USA), ethanol (99.9%, VWR Chemicals, Radnor, PA, USA), distilled water, and nitric acid (69%, Sigma Aldrich, St. Louis, MO) were mixed with a molar ratio of 1:4:2.5:0.04. The mixture was reacted at 60 °C for 3 h to obtain a per-hydrolyzed TEOS solution. Then, cetyltrimethylammonium bromide (CTAB, 99%, Sigma Aldrich, St. Louis, MO, USA) was dissolved into the per-hydrolyzed TEOS solution to achieve CTAB:(SiO_2_ + Al_2_O_3_) molar ratios of 0.5 and 2, thus obtaining two membranes with different porosity. In the second step, aluminum isopropoxide (AIP, 98%, Sigma Aldrich, St. Louis, MO, USA) was added into the mixture, to achieve 5 mol% of alumina doping. After the AIP fully dissolved, the sol was dip-coated on to a commercial (Pervatech B.V. Rijssen, The Netherlands) α-alumina tubular support (250 × 7 mm) with a γ-alumina intermedia layer (Figure 1a). The membranes were then calcinated at 450 °C for 2 h with heating/cooling rates of about 2 °C/min. The two membranes were labeled as S/O = 2 and S/O = 0.5 according to their CTAB:(SiO_2_ + Al_2_O_3_) molar ratios. We learned from the previous study that, in the range of CTAB:(SiO_2_ + Al_2_O_3_) = 0.5 to 4, the S/O = 2 silica-alumina membrane has the optimum permeability, while the S/O = 0.5 exhibit the optimum selectivity [22]. Membrane cross-section was observed over a scanning electron microscope (SEM) EVO 50 XVP microscope (Zeiss, Köln, Germany). The samples were coated with a gold layer (thickness ~25 nm) by a sputter coater (Baltec SCD 050, Pfäffikon, Switzerland) to avoid any charging effect.

### 2.2. Filtration Experiments

Filtration experiments were conducted on S/O = 2, S/O = 0.5, and on the reference NF90 in Figure 1b (FilmTec™ membranes, Dow Chem., filtration area 75 × 58 mm). The three membranes were placed in different housings according to their geometries and tested in a cross-flow nanofiltration apparatus, which is described elsewhere [13]. In brief, the setup consists of an NF module connected with a high-pressure pump which circulates the feed into the system. Two pressure transmitters (Danfoss, MBS 4010, Nordborg Denmark) are present at the module inlet and outlet to measure the corresponding pressures. The permeate is collected into a container placed on a balance to measure its weight. The apparatus was operated at a transmembrane pressure difference of 5.0 bar, with a pumped water flux of around 4 × 10^−6^ m^3^ s^−1^. The membrane permeability was measured by a balance placed below the permeate tank. In each filtration test, the apparatus was fed with 2.0 L of groundwater collected from a well, which is the property of Acquedotto Pugliese S.P.A. (Puglia, Italy). This water sample will be hereinafter referred to simply as “the groundwater”. The membranes were flushed bydemineralized water for 2 h before each filtration experiment. To test the ability of the membrane to retain potential organic contaminants, groundwater samples were sparked with 10 ppm of three model pesticides, namely acetamiprid (ACE, 98%, Sigma Aldrich St. Louis, MO, USA), imidacloprid (IMI, 98%, Sigma Aldrich, St. Louis, MO, USA), and thiacloprid (THI, 98%, Sigma Aldrich, St. Louis, MO, USA).

The specific energy consumption (SEC, kWh⋅m^−3^), i.e., the energy required to produce each m^3^ of freshwater, was estimated by applying Equation (1) to the filtration parameters.

(1) $$SEC=2.778\cdot 10^{-7}\frac{\Delta P\cdot Q_{f}}{Q_{p}}$$

where Δ*P* (here expressed in N m^−2^) is the pressure drop of the feed after passing the membrane module, 2.778 × 10^−7^ is the conversion factor from joule to kilowatt-hour and *Q_f_* and *Q_p_* are volumetric flow rates of feed and permeate streams, respectively. *Q_f_* for all the tested membranes was the same and was equal to 3.83 × 10^−6^ m^3^/s.

### 2.3. Characterization of the Water Sample

The permeate conductivity was measured with a MeterLab (CDM210). The measurements of the permeate mass and conductivity were automatically registered via a MatLab 9.7 (MathWorks, Natick, MA, USA). The concentration of relevant cations was measured by inductively coupled plasma spectroscopy (ICP) (PerkinElmer^®^ Optima 8000 Optical Emission Spectrometer, Waltham, MA, USA) after calibration with standards from PlasmaCAL Q.C. No 4 (SCP Science, Clark, QC, Canada). The concentration of organic pollutants was investigated over a high-performance liquid chromatography (HPLC) apparatus equipped with a Dionex ASI-100 (Phenomenex, Torrance, CA, USA) and a Luna 5 U C18 column (Phenomenex, Torrance, CA, USA). The mobile phase consisted of a water/acetonitrile mixture with ratios of 60/40, 70/30, and 60/40 for acetamiprid, imidacloprid, thiacloprid respectively. The elution rate was set at 1 mL min^−1^. The rejection of the ions and micropollutants was defined according to Equation (2). To evaluate the sodium hazard of the permeate water for irrigation purposes, sodium adsorption ratio (SAR) was applied as Equation (3), where the concentration of the Na^+^, Ca^2+^, Mg^2+^ were expressed as *mEq ^−1^*.

(2) $$Rejection\left(\%\right)=\left(1-\frac{C_{permeate}}{C_{Feed}}\right)\cdot 100$$

(3) $$SAR=\frac{Na^{+}}{\sqrt{\frac{{\mathrm{Ca}}^{2+}+{\mathrm{Mg}}^{2+}}{2}}}$$

## 3. Results

### 3.1. Membrane Structure

**Figure 2:**
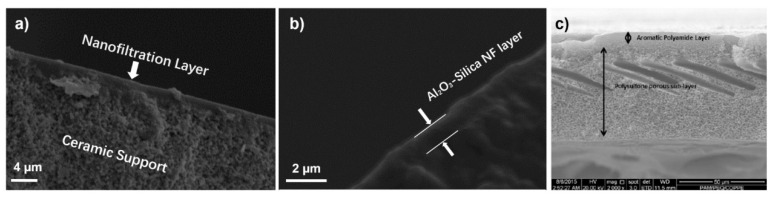
SEM micrographs at (**a**) low and (**b**) high magnification of the cross-section of S/O = 2, and (**c**) cross-section of NF90 (image reprinted from Ref [27] with permission from Elsevier, 2018).

The two Al_2_O_3_-doped silica membranes reported in this study, namely S/O = 0.5 and S/O = 2, were coated from sols with the same Al_2_O_3_ + SiO_2_ loading (6.5 g L^−1^), but a different surfactant/oxide (S/O) molar ratio. From the previous study we found that among the Al_2_O_3_ doped silica membranes with different surfactant concentrations, the S/O = 0.5 has the highest selectivity while the S/O = 2 has the highest permeability [22]. It is not surprising that their final consolidated NF layers have similar structures, but different porosity. Indeed, the Al_2_O_3_-doped silica membranes have an asymmetric architecture (Figure 2a,b), which resemble that of the commercial polymeric NF90 (Figure 2c). The thickness of the Al_2_O_3_-doped silica selective layers is about 0.8 µm, but due to the different concentrations of the surfactant, the pores of S/O = 2 are more interconnected than S/O = 0.5. Nevertheless, according to the low-temperature N_2_ adsorption experiment from the former study, the pore size of the two membranes is similar at around 1−2 nm, and the specific surface area was at 695 and 685 m^2^/g for the S/O = 0.5 and S/O = 0.5, respectively [22]. On the other hand, NF90 has a smaller pore size (around 0.55 nm) and the thickness of the active layer at around 0.29 µm.

### 3.2. Water Permeability

**Figure 3:**
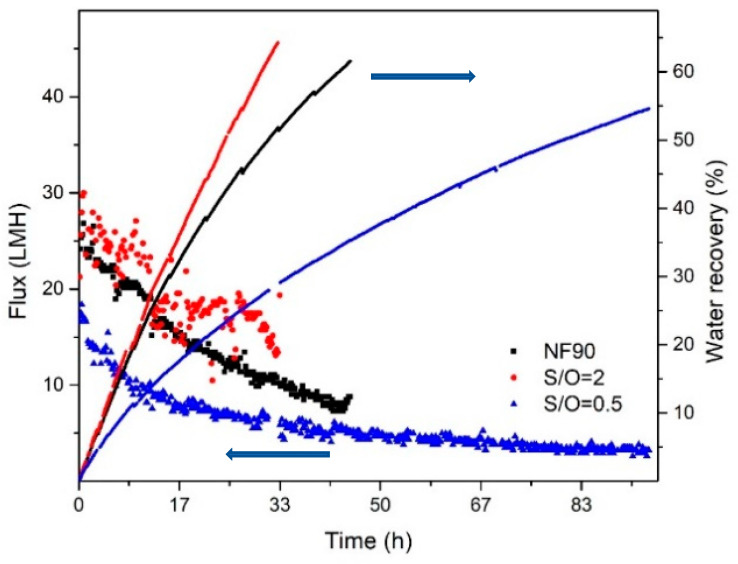
Comparison of the fluxes and water recovery factor as a function of the time for the polymeric commercial NF90 and the lab-made Al_2_O_3_-doped silica membranes S/O = 2 and S/O = 0.5, when filtering 2.0 L of groundwater.

**Figure 4:**
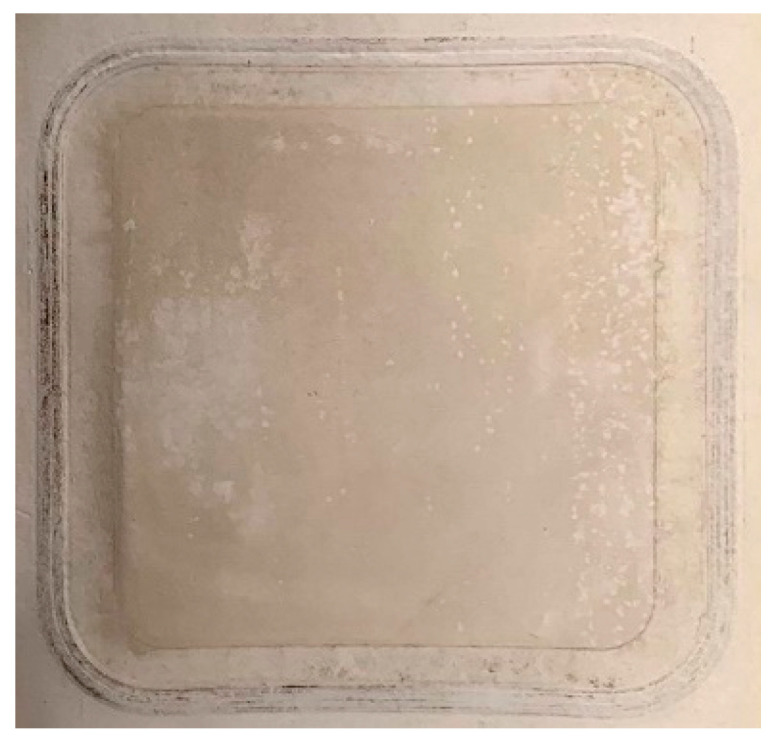
The surface of NF90 after filtering groundwater (final recovery factor = 60%).

Figure 3 shows permeate fluxes and recovery factors of the NF90 and the two Al_2_O_3_-doped silica membranes, namely S/O = 0.5 and S/O = 2 when filtering the groundwater (initial volume 2.0 L) at 5 bar of transmembrane pressure. At 1% of water recovery, the permeates fluxes for S/O = 2, NF90, and S/O = 0.5 were at 28, 25, and 17 LMH (i.e., L·m^−2^·h^−1^), respectively. However, when the recovery factor reaches 50% (i.e., after collecting 1.0 L of permeate), the flux reduces to 19, 11, and 3 LMH for S/O = 2, NF90, and S/O = 0.5, respectively. For S/O = 0.5, the flux becomes relatively stable after 50 h, which corresponds to a recovery factor of 38%. The decreasing trend of the flux for all three membranes could be attributed to two factors: the increase of osmotic pressure caused by the increasing feed concentration during the filtration; and the formation of the fouling/scaling layer on the surface of the membrane (Figure 3). We assume the anions present in the feed water were HCO_3_^−^, Cl^−^ and SO_4_^2−^. Base on charge balance, a rough estimation of the osmotic pressure of the initial feed and after treatment can maximum account for a flux reduction of 30% for the NF90 membrane, whereas the experimental data shows a flux reduction of around 64%. Therefore, fouling and/or scaling are expected to play a more prominent role in the flux decline during filtration. This was confirmed by visually inspecting the surface of the membranes after the filtration tests. Indeed, as shown for NF90 in Figure 4, where a layer of deposits is clearly visible at the membrane surface. 

Overall, the S/O = 2 membrane has the highest flux. This can be the result of the high pore density of the membrane when a large amount of surfactant was added into the coating-sol during the membrane synthesis. The three membranes were operated to reach a final water recovery factor of about 60%, which could be easily achieved in our nanofiltration apparatus. Slightly different recovery factors (55–65%) for each membrane were observed in the study, this could due to the different operating times with the three membranes (Figure 3). Similar to the fluxes, the recovery rate was decreasing overtime for all three membranes.

### 3.3. Ionic Selectivity

**Figure 5:**
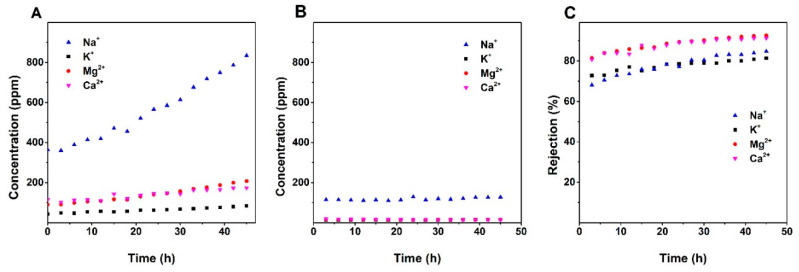
(**a**) Change of cations concentration of the feed and permeate (**b**) over time; (**c**) the change of ions rejection for the polymeric membrane NF90 over time.

**Figure 6:**
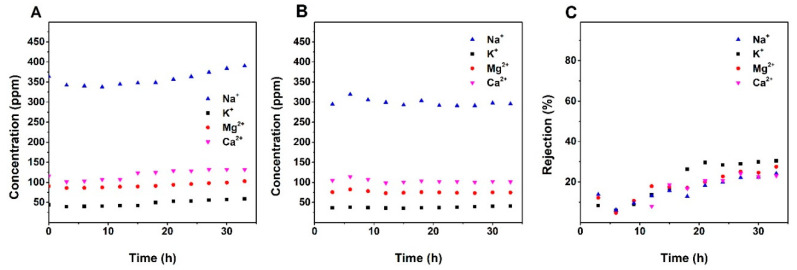
(**a**) Change of cations concentration of the feed and permeate (**b**) over time; (**c**) the change of ions rejection for the S/O = 2 over time.

**Figure 7:**
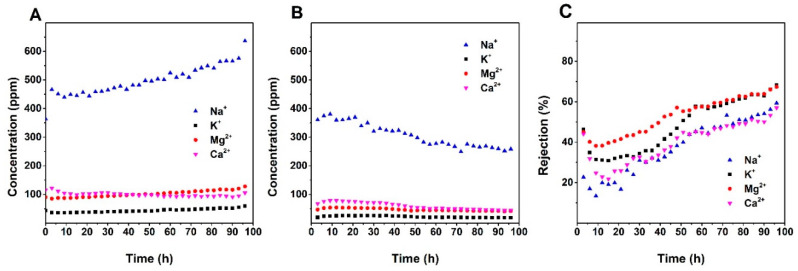
(**a**) Change of cations concentration of the feed and permeate (**b**) over time; (**c**) the change of ions rejection for S/O = 0.5 over time.

The ion selectivities of the membranes were confirmed by the ICP measurement. Figure 5, Figure 6 and Figure 7 show the variation of the concentrations of the major cationic components of the groundwater (i.e., Na^+^, K^+^, Ca^2+^, and Mg^2+^) in the feed and permeate as a function of the time and ion rejections calculated by using Equation (2). Counterintuitively, for all membranes, the cation concentration of the feed was increasing over time while the ion concentration of the permeate kept constant or decreasing during the filtration. This leads to an increasing rejection over time (Figure 5, Figure 6 and Figure 7). The increasing of the NF membrane selectivity during filtration have been reported by several studies, the explanation could be the formation fouling/scaling layer from the deposition of multivalent cation compounds or organic matters on the membrane surface has an enhancement to the selectivity [28,29]. 

A comparison of data provided in Figure 5, Figure 6 and Figure 7 shows that NF90 has the highest ion rejection among all tested membranes. After 45 h of filtration, the rejection for Mg^2+^ and Ca^2+^ was up to around 90%. NF90 has shown a slightly higher rejection for divalent ion than monovalent ions, this is typical for the polymer NF membranes since the membrane is more efficient at retaining hydrated ions with bigger diameter and higher charge, due to the size exclusion and electrostatic force [30]. The inorganic membranes showed a lower rejection compare to the NF90. By the end of the filtration, the rejection of Mg^2+^ and Ca^2+^ were at around 67% and 57% for S/O = 0.5, and at around 28% and 23% for S/O = 2. This number is much lower compare to the data obtained from the previous study, where the highest rejection for the divalent ions was above 90% [22]. The possible explanation for the relatively low rejection could be the high ion concentration in the groundwater compare to the model solutions tested in the previous study.

### 3.4. Water Potabilization

**Figure 8:**
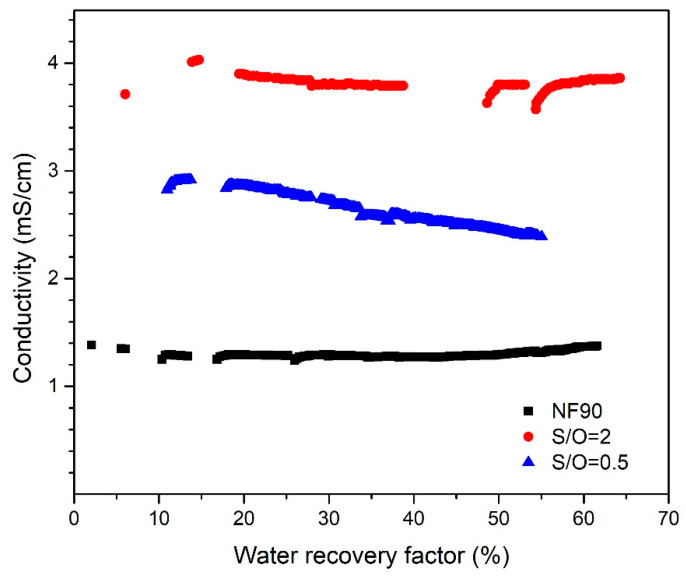
The conductivity of the permeates for the polymeric Dow 90 NF and the inorganic membranes S/O = 2 and S/O = 0.5 as a function of the water recovery factor.

The permeate conductivity for each membrane is reported as a function of the water recovery factor in Figure 8. Despite the increase of the ion concentration in the feed over the water recovery factor, the conductivity kept relatively constant for all the membranes. This indicates that a stable performance can be obtained for all three membranes throughout the filtration experiment. When the water recovery factor reaches 50%, the permeates conductivity for S/O = 2 and S/O = 0.5 was observed at around 3.8 mS·cm^−1^ and 2.4 mS·cm^−1^ respectively. At the same water recovery factor, the lowest permeate conductivity was obtained by NF90 at around 1.3 mS·cm^−1^, which is significantly lower than the conductivity of the groundwater sample (4.6 mS·cm^−1^). The permeate conductivity for the membranes was consistent with the ICP measurements that showed in Figure 5, Figure 6 and Figure 7. The conductivity level of the NF 90 permeate has reduced to almost half of the conductive limit (2.5 mS·cm^−1^) according to the Italian standard [26], thus, it is safe to assume the NF90 permeate suitable for human consumption. In principle, S/O = 0.5 can also produce drinking water from the groundwater sample treated in this study. Nevertheless, fluctuations in the feed salinity might results in a permeate with conductivity higher than 2.5 mS·cm^−1^ and therefore not suitable for human consumption. As for the S/O = 2, the permeate has a conductivity largely above the limitation, therefore it is not recommended for consumption as well.

On the other hand, the permeate water could also potentially be used for irrigation purposes since two major concerns for irrigation water is the salinity hazard and sodium hazard, which are generally indicated by the water conductivity and SAR (Equation (3)), respectively. For the conductivity, in the range between 0.76–2.0 µS·cm^−1^, depending on the species of the plants, the permeate water of NF90 could be used for irrigation of plants with a moderate salinity tolerance, such as tomatoes, soybeans, and wheat [31]. Additionally, the permeate water of S/O = 0.5 is in the range between 1.5 to 3.0 mS·cm^−1^, which could be used for the irrigation of plants with a high salinity tolerance like cotton or wheatgrass [31]. On the other hand, SAR can be used to measure the risk of the irrigation soil subject to sodium hazard, since the presence of Na^+^ could be harmful to the plants. The SAR value for the permeate of NF90 and S/O = 2 were in the range of 4.5 to 5.5, whereas the SAR value for the permeate water of S/O = 0.5 was in the range of 6 to 8. According to the Food and Agriculture Organization (FAO), all the permeate has a SAR below 9, therefore the soil for irrigation subject to no or little sodium hazard [32,33]. It is also worth mentioning that minerals such as calcium, potassium, and magnesium, are fertilizers for plants and their controlled inclusion in water can have a beneficiary effect upon the crops and vegetable production. Thus, the water produced through NF in the current study, can also partially fulfill the fertilizer needs of the plants.

**Figure 9:**
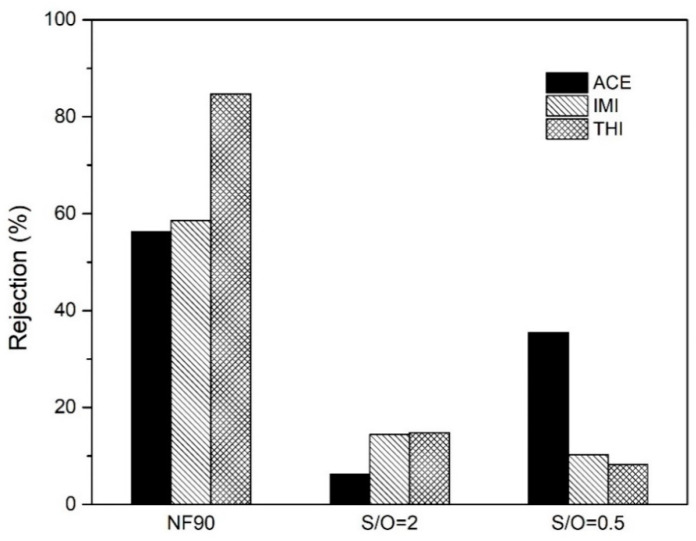
Rejection of acetamiprid (ACE), imidacloprid (IMI), and thiacloprid (THI) for the commercial polymer membrane (NF90) and the Al_2_O_3_-doped silica membranes (S/O = 2 and S/O = 0.5).

No harmful concentration of organic micropollutants was found in the groundwater sample. Nevertheless, we sparked a groundwater sample with three model pesticides (10 ppm of ACE, IMI, and THI) to mimic a case in which the membrane feed is contaminated with micropollutants and thus to fully investigated the potential of the NF membranes in water potabilization. The rejections of the micropollutants for the membranes is depicted in Figure 9. For the ion rejection, among the three membranes NF90 is the one with the highest rejection for ACE, IMI, and THI: 56%, 59%, and 85% respectively. The inorganic membranes showed a lower selectivity. The rejection of the ACE, IMI, and THI for the S/O = 0.5 was at 35%, 10%, and 8%, respectively, and 6%, 14%, and 15% for S/O = 2. It is clear to see from the data above that the NF90 has a better perm/selectivity among all membranes. 

### 3.5. Specific Energy Consumption 

**Figure 10:**
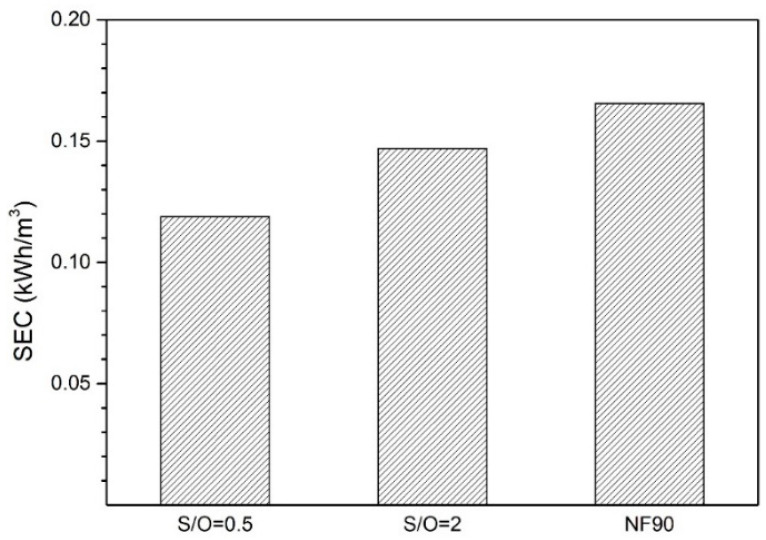
Specific energy consumption of freshwater produced by using different membranes considered in the current study.

Figure 10 reports the specific energy consumption (SEC) for the three membranes, as calculated from Equation (1). The observed pressure drops for S/O = 2, S/O = 0.5, and NF90 were 0.014, 0.003, and 0.01bar, respectively whereas, the corresponding permeate flow rates, *Q_p_*, were 1.05 × 10^−8^, 3.24 × 10^−9^ and 7.41 × 10^−9^ m^3^/s, respectively. It is evident from the figure that S/O = 0.5 demonstrates the minimum SEC among the tested membranes. *Q_p_* for NF90 and S/O = 0.2 are, respectively, 56 and 69% higher than S/O = 0.5, however, the corresponding pressure drops for these membranes are even higher (70 and 78.5%, respectively). Consequently, S/O = 0.5 demonstrates the minimum SEC. NF90 is the most energy-consuming membrane among all the tested membranes due to its mediocre flux and relatively high pressure drop and demonstrates almost 1.4 times higher SEC than S/O = 0.5. The SEC values observed in the current study are similar or even slightly lower than what has been reported in the literature for similar feed water composition [34,35], thus indicating a good perspective of the applied Al_2_O_3_-doped silica membranes in desalination through NF. State-of-the-art polymeric nanofiltration membranes are in flat sheet configuration and require the use of spacers to support the membranes as well as to alleviate the concentration polarization within the module. The presence of spacers, however, causes additional pressure drop within the membrane module. Tube-shaped Al_2_O_3_-doped silica membranes used in the current study do not require any spacers and therefore, pressure drop within the module channels remains low. Relatively lower energy consumptions for ceramic membranes observed in the current study can be attributed to lower pressure drop compared to traditional flat sheet polymeric membranes where the applied spacers contribute significantly in total observed pressure drop [36]. 

Considering the energy cost of 0.15 €/kWh for industrial users in Italy [37], SEC discussed in the above paragraph translate into specific water cost between €0.018–0.024 for each cubic meter of the freshwater obtained. The specific cost for commercial freshwater in the Puglia region is 1.5 €/m^3^ which indicates that NF is an attractive option for the production of fresh water from underground water in the region. 

## 4. Conclusions

A commercial polymer membrane and two lab-made inorganic membranes were tested for the desalination of groundwater from a well in the Puglia Region, Italy. Among the three tested membranes, the polymer membrane, NF90, have shown promising performance regarding the selectivity and permeability, with around 80–90% rejection for divalent ions, and 56–85% for micropollutants. From the filtration experiment, around 62% of water can be recovered, the recovered water from NF90 can be potentially used for human consumption or irrigation. On the other hand, the inorganic membranes S/O = 2 and S/O = 0.5 have shown a lower selectivity, the permeate conductivity was 3.8 mS/cm and 2.4 mS/cm, respectively. Due to the high salinity of the permeate water for both inorganic membranes, it is not recommended for drinking. However, the permeate water of S/O = 0.5 could potentially be used for the irrigation of plants with high salinity tolerance. In terms of energy consumption, S/O = 0.5 demonstrated the lowest SEC among the tested membrane which was equivalent to a specific water cost of around 0.02 €/m^3^. The specific water cost for the ceramic NF membranes, observed in the current study, is less than 3% of the commercial price of freshwater in the region that demonstrates the excellent economical potential of NF for the treatment of underground water in the region.
